# Dissolving Microneedle Patches for Dermal Vaccination

**DOI:** 10.1007/s11095-017-2223-2

**Published:** 2017-07-17

**Authors:** M. Leone, J. Mönkäre, J. A. Bouwstra, G. Kersten

**Affiliations:** 1 Division of Drug Delivery Technology, Cluster BioTherapeutics, Leiden Academic Centre for Drug Research, Leiden University, Einsteinweg 55, P.O. Box 9502, 2300 RA Leiden, the Netherlands; 2 Department of Analytical Development and Formulation, Intravacc, Bilthoven, the Netherlands

**Keywords:** antigen stability, dissolving microneedle fabrication, dissolving microneedle characterization, skin immunization, vaccine delivery

## Abstract

The dermal route is an attractive route for vaccine delivery due to the easy skin accessibility and a dense network of immune cells in the skin. The development of microneedles is crucial to take advantage of the skin immunization and simultaneously to overcome problems related to vaccination by conventional needles (e.g. pain, needle-stick injuries or needle re-use). This review focuses on dissolving microneedles that after penetration into the skin dissolve releasing the encapsulated antigen. The microneedle patch fabrication techniques and their challenges are discussed as well as the microneedle characterization methods and antigen stability aspects. The immunogenicity of antigens formulated in dissolving microneedles are addressed. Finally, the early clinical development is discussed.

## Introduction

**Fig. 1:**
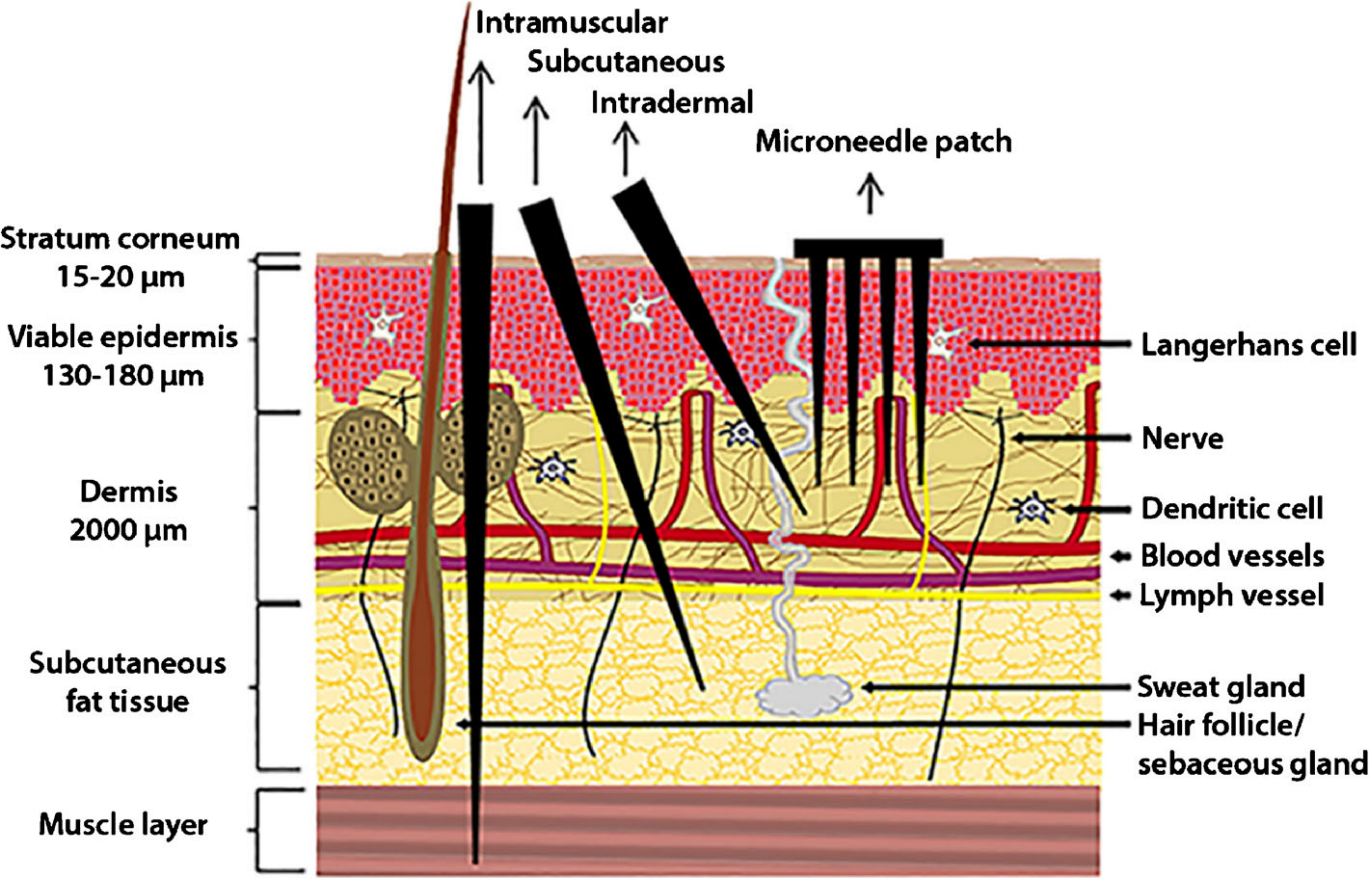
Schematic representation of microneedle insertion and conventional (intramuscular, subcutaneous and intradermal) injections onto the human skin are shown. Microneedles penetrate the stratum corneum reaching the viable epidermis. The hypodermic needles puncture the skin during insertion into the subcutaneous or muscle tissues. Adapted from (3).

Vaccination is one of the most successful medical interventions in history, reducing mortality and morbidity for several infectious diseases to almost zero in areas where vaccines are being used (1,2). Most vaccines are administered intramuscularly or subcutaneously (Fig. 1) by injection that may cause pain and discomfort and avoidance by people with needle-phobia (4–7). Furthermore, the hypodermic needles used to administer the vaccine by these routes generates hazardous waste and can lead to needle stick-injuries and needle re-use. The latter can spread infectious diseases such as Hepatitis B and AIDS particularly in the developing countries (8). Furthermore, the use of innovative vaccine delivery systems could offer several other advantages such as antigen thermostability, fewer booster immunizations and, as a consequence, increase of the vaccination adherence and a reduced burden on healthcare personnel. These latter advantages would especially be beneficial in mass vaccination campaigns, such as in case of outbreaks, when feasible and fast immunizations schemes are necessary (4).

Since the skin is a very immune-competent organ and easily accessible, dermal vaccine delivery is an attractive alternative. The viable epidermis and dermis contain many antigen presenting cells (APCs) such as Langerhans cells (LCs) and dermal dendritic cells (dDCs) (Fig. 1) (9,10). These APCs capture antigens and subsequently migrate to the draining lymph nodes to present the antigen to the T-cells to activate Ag-specific T-cells and B-cells for systemic immune response. Besides LCs and dDCs, epidermal keratinocytes are also involved in the immune response by producing cytokines and chemokines (e.g. TNF-α and IL-1β) to enhance maturation of APCs and migration to the lymph nodes (11).

Although the skin surface is easily accessible, the skin (Fig. 1) is designed to protect the human body against entry of foreign organisms or toxic substances (3,12). Therefore, the top-layer of the skin, the stratum corneum (in humans 15–20 μm thick), forms a significant physical barrier for vaccine delivery. Consequently, the delivery of high-molecular weight (>500 Da) compounds such as antigens require methods enabling their penetration into the skin (13). Several methods such as powder and fluid jet injection, thermal microporation, sonoporation, transfollicular delivery and microneedles (9) have been proposed to deliver antigens into the skin. Recently, microneedles (MNs) have gained great attention for dermal vaccination. MNs are needle-like microstructures, up to 1 mm in length (3), typically assembled in variable numbers on a patch. They pierce the stratum corneum and underlying tissue to deliver the antigen into the epidermis or dermis while they are short enough not to reach pain receptors and thus pain sensation can be avoided (7). Furthermore, the immunization with MNs may not require the healthcare personnel (5,6,11) and does not generate sharp needle wastage after immunization.

The first microneedles were conceptualized for drug delivery in 1976 (14) but only during the last 20 years microneedles have been actively developed. MNs can be classified in the following groups: hollow, coated, porous, hydrogel-forming, dissolving microneedles (dMNs) and MNs for pretreatment (15–18). dMNs consist of fast-dissolving materials (e.g. polymers or sugars) as a matrix material and the drug/antigen is mixed in the matrix. After insertion into the skin, they dissolve releasing simultaneously the active pharmaceutical ingredient (3,6,15,16,19).

The scope of this review is to evaluate the use of dMNs as vaccine delivery systems to overcome the limitations of traditional subcutaneous (s.c.), intramuscular (i.m.) or intradermal (i.d.) injections. Preparation methods for dMNs, their characterization and immunological properties will be described underlining the potential and novelty of this new micro-technology.

## Materials and Manufacturing Methods

### Materials

Matrix material should possess the following characteristics: biocompatible, biodegradable, low toxicity, strength/toughness and cheap (17,20). Many materials have been used to produce dMNs (Table I). Head to head comparisons of the materials used for dMN production have not been reported as far as we know. The selection of the matrix material may be based on practical considerations rather than rational design. Apart from safety, factors to consider include obtaining MNs capable to pierce the skin, compatibility with the active compound, compatibility with the manufacturing procedure (acceptable viscosity before drying or spraying and reasonable solidification time) and a potential to scale-up of dMN patches for mass production (16). The most frequently used matrix materials are sodium hyaluronate, that is naturally present in the skin, and sodium carboxymethylcellulose (21–23,31–33). Both are approved as inactive materials by FDA for parenteral drug products. Other materials include poly(vinylalcohol) (PVA) (42), poly(vinylpyrrolidone) (PVP) (43), methylvinylether-co-maleic anhydride (PMVE/MA) (Gantrez AN-139®) (44,45) and low molecular weight sugars like maltose (46,47) and trehalose (48). dMNs have also been prepared from biodegradable polymers such as polylactic-co-glycolic acid (PLGA) (46), polylactic acid (PLA) (49) and polyglycolic acid (PGA) (50). However, due to their slow dissolution rate in skin and a preparation method using high temperatures (34) and organic solvents, these polymers are less suitable as matrix material. The back-plate of the dMN patch can be made by using the same (51) or different materials (30,42) as the needles. Furthermore, the back-plate can be reinforced or the ease of handling can be increased by applying an adhesive tape (38,42,52–54). Besides matrix material, other excipients might be included ((33) (32) (30)) to improve the antigen stability or mechanical strength of the dMNs (Table I).

**Table I:** Overview of Matrix Materials and Antigens Used for dMN Vaccination Studies. Back-Plate Materials are Not Listed in this Table

dMN composition	Antigen (Ag)	Adjuvant (Adj)	Ref.
Sodium hyaluronate	OVA		(21)
Sodium hyaluronate	Adeno virus		(21)
Sodium hyaluronate, dextran 70 and polyvidone	TT/DT		(22)
Sodium hyaluronate	TT/DT		(23)
Sodium hyaluronate	SE36 recombinant molecule (malaria vaccine)		(23)
Sodium hyaluronate	Trivalent influenza		(23)
Sodium hyaluronate	EV71 virus-like particles		(24)
PVP	OVA	CpG OND	(25)
		Co-encapsulation in cationic liposome	
PVP	Whole inactivated influenza virus		(26)
PVP	Plasmid vector VR2012 encoding the middle envelope proteins of HBV	CpG ODN	(27)
		Co-encapsulation in cationic liposome	
Gantrez® AN-139	OVA	Encapsulation in PLGA NPs	(28)
Gantrez® AN-139 and polysorbate 80	HIV-1 CN54gp140	MPLA	(29)
Sucrose and threonine	IPV		(30)
Maltodextrin			
Sucrose, threonine and CMC	Live-attenuated measles vaccine		(31)
Na-CMC and trehalose	Monovalent subunit influenza vaccine		(32)
	Trivalent subunit influenza vaccine		
Na-CMC, sucrose and lactose	Adenovirus expressing OVA		(33)
	Adenovirus expressing HIV-1 CN54 gag		
PAA	OVA	Poly(I:C) loaded NPs	(34)
PAA	OVA	Silk depot loading OVA	(35)
		poly(I:C)	
Sodium chondroitin sulfate	OVA		(36)
Chitosan	OVA		(37)
Trehalose and PVA	Inactivated split trivalent influenza vaccine		(38)
Dextran 70 and sorbitol	Trivalent subunit influenza vaccine		(39)
Fish gelatin and sucrose	Subunit monovalent influenza vaccines		(40)
PVA and sucrose	DNA plasmid expressing rabies G protein		(41)

*CpG ODN* CpG oligodeoxynucleotides, *DT* diphtheria toxoid, *EV71* Enterovirus 71, *Gantrez® AN-139* copolymer of methylvinylether-co-maleic anhydride (PMVE/MA), *HIV* human immunodeficiency virus, *HBV* hepatitis B virus, *IPV* inactivated polio vaccine, *MPLA* monophosphoryl lipid A, *NPs* nanoparticles, *Na-CMC* Sodium carboxymethylcellulose, *OVA* ovalbumin, *PAA* poly(acrylic acid), *PLGA* poly-D,L-lactide-co-glycolide, *poly(I:C)* polyinosinic-polycytidylic acid, *PVA* poly(vinylalcohol), PVP poly(vinylpyrrolidone), *TT* tetanus toxoid

Antigens that have been used include almost all vaccine types, ranging from peptides and proteins (21–23) to DNA vectors encoding antigenic proteins (27,33,41) and attenuated or inactivated viruses (26,30,31). Antigens are generally dispersed directly in the dMN matrix (21–23,31,32) but they can also be encapsulated in nanoparticles or in a cross-linked structure (25,28,35) to potentiate or alter the immune response (25,34,35). Furthermore, adjuvant can be incorporated in the dMNs (55).

### Manufacturing Methods

#### Micromolding

**Fig. 2:**
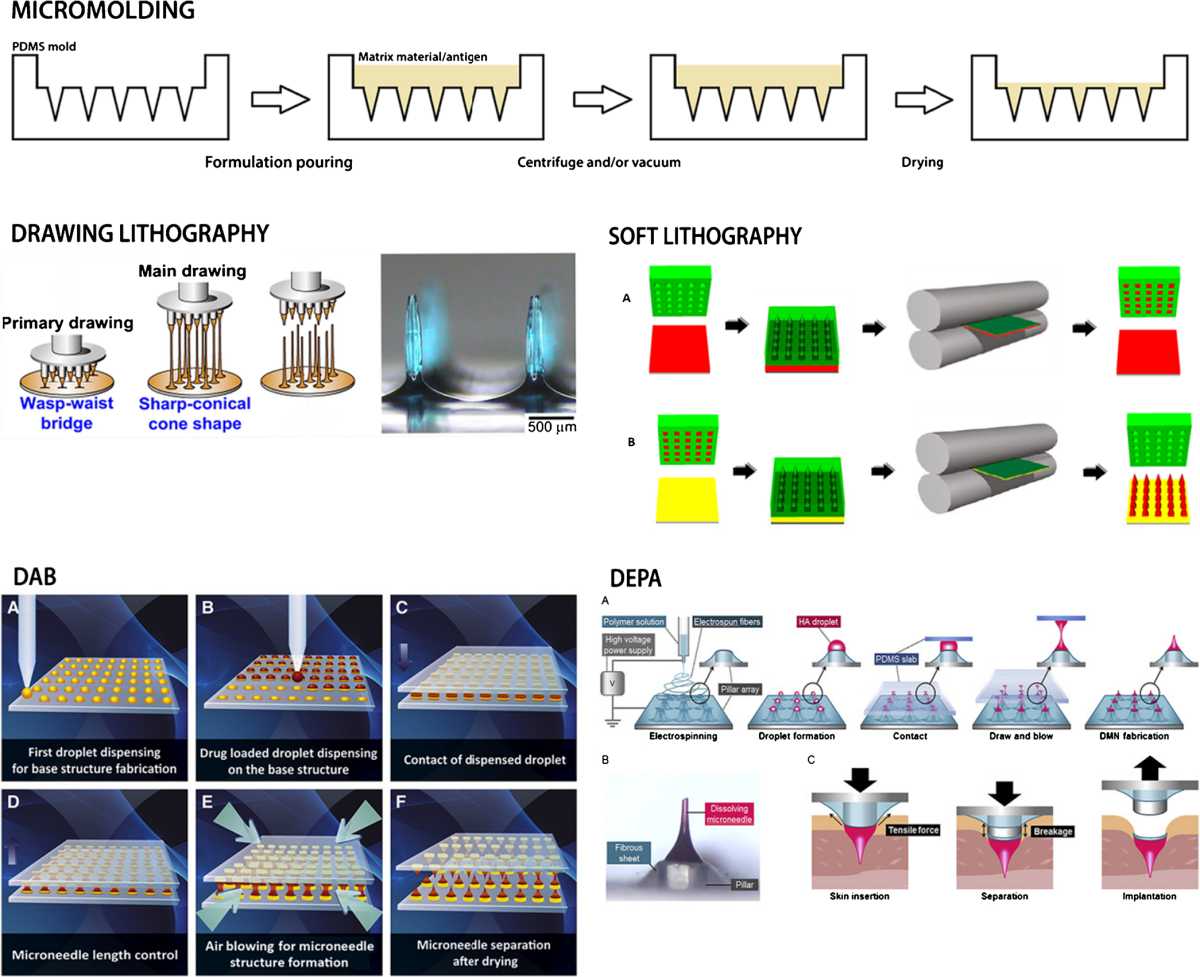
dMN Manufacturing Methods. See main text for details. Adapted from (66–69). DAB = droplet-born air blowing; DEPA = dMN on an electrospun pillar array.

The most common fabrication method of dMNs is micromolding in which dMNs are prepared using a polydimethylsiloxane (PDMS) mold (Fig. 2). First, the PDMS mold is typically produced from a silicon or metallic master mold (17) that is obtained by using techniques such as etching (56), lithography (57), thermal drawing (58) and laser micromachining (59,60). PDMS is a hydrophobic flexible material, which can very accurately reproduce the master structure as a negative template (17). The mold can be re-used for dMN fabrications after appropriate cleaning. The first step in preparing dMNs using the PDMS mold is the addition of the polymer/antigen mixture in the mold. This is typically done manually at the research setting but the mold can also be filled by using an atomized spray (48). After filling of the mold, vacuum and/or centrifugation steps are performed to fill the PDMS microcavities with the polymer/antigen mixture (61). Finally, the solution in the mold is dried at slightly elevated temperature (62,63). The drying step can be replaced by photopolymerization if photocrosslinkable material is used (60).

The micromolding can be a straightforward technique in the laboratory because it requires little additional equipment. Furthermore, the absence of harsh conditions (e.g. high temperature or organic solvents) is an advantage when working with sensitive antigens (64). However, it might not be suitable for industrial scale-up or continuous manufacturing if steps such as manual removal of air bubbles from the microcavities after vacuum or centrifugation are needed or if the production method will result in too much vaccine wastage (see Antigen Wastage section).

#### Lithography

##### Drawing Lithography

This technique is based on extensional (stretching) deformation of polymeric material from a 2-dimensional to a 3-dimensional structure. Melted polymer is dispensed on a fixed plate and elongated by drawing pillars in the upper-moving plate (Fig. 2) (65,66). The polymer viscosity is progressively increased by cooling until the glass transition temperature of the polymer is reached. Finally, further cooling induces a solid polymer providing the suitable dMN strength for the skin piercing (19,66). The advantage of this fast fabrication method is the minimal polymer wastage due to the dispensed drops on the plate. However, only a limited number of polymers have suitable glass transition temperatures for this method (65). More importantly, this technique is not appropriate for thermolabile antigens because melting and transition temperatures are high during the manufacturing (e.g. for maltose >95°C (66)).

##### Soft Lithography

In soft lithography dMNs are fabricated by first pairing a polymer film with the mold with microcavities and passing them through a heated nip. Next, the filled mold is placed on a flexible, water-soluble substrate and passed through the heated nip. After separation of the mold, a dMN patch on the substrate remains (Fig. 2). Instead of heated nip, photocuring can be also used (67). Similarly to drawing lithography, this manufacturing method claims excellent scalability, low cost and short preparation time. However, the high temperature used for the fabrication can be still critical while using a thermolabile antigen mixed with the matrix.

#### Droplet-Born Air Blowing and dMN on an Electrospun Pillar Array

In droplet-born air blowing (DAB), a droplet of polymer solution without drug and another droplet of drug solution are dispensed together on two plates. The upper plate is moved downwards so that the droplets are touching and thereafter plates are withdrawn to a distance corresponding to the two dMN lengths of the lower and upper plate (Fig. 2). The polymer solutions are dried with air flow producing a dMN patch on each plate (Fig. 2) (68). The advantages include low temperature (4–25°C) and fast (≤ 10 min) fabrication and minimal drug and polymer wastage.

A variant of DAB is dMN on an electrospun pillar array (DEPA). The flat plate is replaced here by a pillar array covered by a fibrous sheet. Then, polymer formulation droplets are dispensed on the pillar array and placed in contact with a PDMS slab to pull and elongate the droplets obtaining microneedles (Fig. 2). Finally, elongated droplets are dried by air flow.

### General Challenges of dMN Preparation

#### Antigen Wastage

Dermal vaccination is attractive especially for the antigen dose sparing to evoke an immune response. However, the optimization of the manufacturing methods is crucial to reduce antigen wastage. During micromolding part of the antigen is lost in the PDMS mold due to low volume filling of the microcavities relative to the system volume needed (70). It is often mentioned that excess of solution from the mold can be collected in order to recycle (35,51,63). However, the saved antigen amount is often not reported in the literature and more importantly the quality of the recovered antigen may be difficult to guarantee hampering reuse of the vaccine formulation.

One possibility to reduce the antigen loss during the micromolding is to use polymer/antigen solution only for the dMNs and to produce a backplate only from the matrix material or even from other material. The backplate material should possess higher viscosity than that of the needles to reduce the diffusion of the antigen from the dMNs during preparation and drying (51). In stability studies presence of antigen in the needles and its absence in the backplate should be monitored to demonstrate lack of diffusion of antigen to the backplate during storage (62). However, in literature this aspect is generally not addressed. In fabrication methods like drawing lithography, DAB and DEPA, the antigen is dispensed in drops, thus the antigen wastage can be potentially reduced drastically. However, it is not reported if antigen can be lost in the dispensing instrument.

#### Antigen and Adjuvant Loading

Besides reproducible loading (61) and dose homogeneity, dMNs should contain a sufficient high antigen and adjuvant dose, which can be challenging due to very low volumes of dMN tips. This can be particularly challenging in the case of antigens encapsulated in nanoparticles, an approach to improve immunogenicity of dermally delivered subunit antigens (28). Another aspect to consider is the delivery efficiency, i.e. the relation between the antigen amount incorporated into the dMNs and the antigen dose actually delivered into the skin. Unfortunately, these aspects are often not described in detail in the literature, although systems have been and are in development to maximize delivery (see next section). This makes comparison of different concepts difficult if not impossible. An additional issue is the physico-chemical properties of the adjuvant, that determines whether the adjuvant can be mixed properly with the matrix material.

#### Fabrication Aimed to Improve Delivery Efficiency

In order to facilitate the delivery of the entire intended antigen dose into the skin, some modified fabrications have been developed. These include micromolding of arrowhead dMNs mounted on mechanically strong shafts (37,63) or dMNs presenting an elongated base increasing the needle length (51). Drawing lithography has been modified by dispensing melted polymer on a fixed plate presenting pedestals (71). DEPA presents patch pillars to improve the delivery efficiency. After patch application into the skin, dMNs separate from the pillars due to a tensile breaking force of the fibrous sheet between the pillar and the dMN (Fig. 2). This allows a proper implantation of the dMNs into the skin and removal of the remaining back plate without the need to wait dMN dissolution (69).

#### Antigen Degradation

Other critical steps during the dMN preparation are related to the high temperature reached in some manufacturing methods. The micromolding usually is done at mild temperatures. However, when using methods such as drawing and soft lithography, temperatures around 100°C may be required. Such a temperature can be critical when using thermolabile antigens mixed to the matrix. Alternatively, photocurable polymers like acrylate-based polymers (72) and poly (ethylene glycol) diacrylate (PEGDA) (73–75) may be used. However, radiation should not damage proteins or DNA of vaccines. In all methods, a drying step is included which can be detrimental for protein antigens even at moderate temperatures (76).

#### Sterility

Because dMNs deliver antigen into the viable skin, they should be sterile and have low endotoxin content (77). Since the final product is dry, a sterile filtration step, if at all possible, should be done on final fluid bulk, implying that the actual patch manufacturing should be performed under aseptic conditions (77). Alternatively, sterilization of patches by gamma irradiation may be considered, although this can damage the antigen (77) and may be difficult to validate. Based on FDA guidelines for medical devices in direct contact with lymphatic tissue, the endotoxin content in dMNs should be <20EU/device (78).

## Characterization of Dissolving Microneedles

A number of unique parameters must be determined to assess dMN quality (Table II). Since there are no licensed products on the market, no MN monographs exist in pharmacopoeias (79). Below, aspects that may be of importance are discussed.

**Table II:** dMN Characterization Methods

Characteristic	Characterization method
Appearance	Microscopy techniques
Antigen distribution in MNs	Confocal microscopy
Water content	Thermogravimetric analyser
	Karl Fischer
	Moisture balance
Antigen stability	Immunogenicity
	Antigenicity: ELISA, SRID, virus titration
	Physico-chemical characterization: intrinsic fluorescence, CD, SDS-PAGE
	Aggregation: HP-SEC, NTA, MFI, AF4, TEM, DLS
Mechanical strength	Displacement-force test station
Skin piercing efficiency	Skin staining and histological sections
Dissolution of MNs	Dissolution of MNs *in vitro*
	Change in MN tip length after skin insertion
Antigen localization into the skin	Microscope analysis of skin sections or confocal microscopy analysis of intact skin
	Analysis of histological skin sections
Antigen quantification	Quantification of antigen concentration after *in vitro* dissolution of dMNs by suitable methods (e.g. UV-vis, fluorescence or ELISA)
	Quantification of antigen delivered into the skin by e.g. radioactivity or infrared imaging
Stability after storage	Forced (elevated humidity and temperature) and real time stability testing

*AF4* asymmetrical flow field–flow fractionation, *CD* circular dichroism, *DLS* dynamic light scattering, *ELISA* enzyme-linked immunosorbent assay, *HP-SEC* size exclusion chromatography, *MFI* micro-flow imaging, *NTA* nanoparticle tracking analysis, *SDS-PAGE* sodium dodecyl sulfate-polyacrylamide gel electrophoresis, *SRID* single radial immunodiffusion assay, *TEM* transmission electron microscopy, *UV-Vis* ultraviolet–visible spectroscopy

### Appearance

Shape and sharpness of MNs are typically investigated by microscopic techniques such as light and scanning electron microscopy (80–84). During product development, microscopy can be used also to analyze the distribution of fluorescent-labelled antigen in the MNs (61).

### Water Content

dMNs are dry formulations and it is important to measure their water content by using methods such as Karl Fisher titration (a coulorimetric or volumetric titration to determine trace amounts of water in the sample), thermogravimetric analysis or moisture balance (85). The water content can influence mechanical properties, protein stability and dissolution kinetics (81). The generally recommended water content for freeze-dried vaccines is less than 3% (*w*/w) (79), that could be also taken as guideline for dMNs.

### Antigen Stability

Stability of the antigen should be assessed both after the manufacturing of dMNs (86) as well as after the storage (22,24,31,38,39). The type of stability indicating assays depends on the antigen as well as the type of immunity that should be induced (e.g. for antibody responses the tertiary structure of protein is important). Protein conformation can be assessed by spectroscopic techniques such as circular dichroism (80) and fluorescence spectroscopy (61). Protein backbone integrity can be analyzed also by SDS-PAGE (25). However, this method is not suitable to examine the protein unfolding, indicating the loss of B-cell epitopes. The obvious way to analyse B-cell epitopes is by measuring antigenicity with immunoassays such as ELISA. In case of incorporation of DNA in dMNs, agarose gel electrophoresis and *in vitro* transfection can be performed to measure the DNA supercoiling and efficacy respectively (41).

The aggregation of protein antigens or particulate vaccines can be investigated by several methods such as size exclusion chromatography (HP-SEC) (61), asymmetrical flow field–flow fractionation (AF4) (61), micro-flow imaging (MFI) (61), transmission electron microscopy (TEM) (24), dynamic light scattering (DLS) (24) and nanoparticle tracking analysis (NTA). For live attenuated or vector vaccines the viability of virus or bacterium may be sufficient because the antigen will replicate after immunization and so the vaccine potency can be determined by measuring the titer of live antigen (31). Finally, immunogenicity studies are crucial to determine vaccine potency (26). A limiting factor for characterization and quality control may be the small sample sizes and matrix effects due to high concentrations of matrix component after dissolution of the dMNs.

So far, a few studies have systematically analyzed vaccine stability in dMNs. Mistilis *et al*. showed that the buffer composition and preparation conditions (e.g. drying temperature) must be carefully selected to retain the vaccine stability of subunit influenza vaccine (85). ELISA analysis of hemagglutinin activity showed that ammonium acetate buffer (pH 7.0) and HEPES retained the antigenicity much better in solution and dry state than when using phosphate-buffers. In addition, surfactants destabilized the antigen especially in liquid formulation prior to dMN fabrication and they may cause crystallization of the MN matrix damaging the antigen (85). Antigen encapsulation plays also a role in the antigen stabilization. Similar antigen-specific CD8+ proliferative responses for OVA-PLGA NPs in dMNs before and after 10 weeks storage at ambient conditions were obtained (28). In contrast, groups immunized with 10 weeks stored monomeric OVA in dMNs showed a decrease in T-cell response in comparison with the group immunized with non-stored one (28).

### dMN Mechanical Strength and Skin Penetration

The mechanical properties of MNs (e.g. strength or facture force) should be analyzed to determine whether dMNs are strong enough and do not fracture during skin penetration (87), unless it is intended so. Measurements of dMN displacement-force can be performed by using a displacement-force test station to compare different matrix materials or geometry (80,83) or the effect of storage conditions (24). Subsequent skin penetration studies are typically analyzed on *ex vivo* human (61) or porcine skin (88). However, it is also important to consider the *in vitro-in vivo* correlation of the subcutaneous layers as these layers can also affect microneedle performance. For this purpose, artificial gel-layers can be used to resemble the *in vivo* situation more closely (89). After MN application and removal from the skin, the skin is stained with dye (e.g. trypan blue). Additionally, stratum corneum can be stripped and the number of penetrating tips per patch can be determined. The penetration of single MN through the skin layers can be examined in a detailed way by analysing histological cross-sections of skin, although this is a more laborious approach (37,51,80) and not suitable for routine analysis. The depth of deposition of fluorescently-labelled antigen in the skin can be investigated by confocal microscopy (61) or fluorescence microscopy by using skin cryo-sections (24).

### dMN Dissolution

The analysis of the dissolution process of MNs is crucial for reproducible antigen disposition in the skin. The dMN dissolution time can be investigated *in vitro* by immersing MNs in buffer (e.g. PBS) (82). This allows the assessment of the quantity and quality of the dissolved antigen. When focusing on dissolution in the skin, the optimal application time of dMN in the skin can be determined by analyzing MN length after the pre-determined application periods (24,80,81). The dMN dissolution in *ex vivo* skin typically resembles the *in vivo* use of MNs. However, it is important to analyze the dissolution also in preclinical studies and in the early clinical development because temperature and humidity conditions may be difficult to mimic in *ex vivo* conditions. Careful preclinical evaluation does not take away the need to study microneedle dissolution in a clinical setting. The contribution of physiological and mechanical properties of the skin at the application site (e.g. thickness, elasticity, etc) to the dMN dissolution rate and antigen delivery may be substantial and should be investigated in the future. Besides reproducible *in vivo* dissolution the actual dose delivered should be determined. Actual dose delivered can be substantially lower than the theoretical maximal dose since the base of the microneedle has a tendency not to dissolve completely. This is an economical risk. In that respect arrow-shaped microneedles having a smaller base, could have advantages above cone-shaped needles.

### Quantification of Antigen/Adjuvant Dose

#### *In Vitro* Analysis

The quantification of antigen dose in dMNs is often very challenging and it can be done *in vitro* by cutting the dMNs from the baseplate and dissolving them (81) or embedding the dMN patch in parafilm and allow MN tips to dissolve in PBS (90). Then, the antigen quantification can be performed for example by fluorescence (81,90), UV-vis analysis (90) or ELISA. The antigen amount in the dMNs can be also determined by dissolving the entire patch (MNs and back-plate) and calculate the volume of the needles based on the needle dimensions. In this case, a prior analysis should demonstrate homogeneous antigen distribution in the entire patch. However, these *in vitro* techniques are difficult to validate. Furthermore, when using an adjuvant, this should also be quantified to confirm its dose, similarly to antigen.

#### *Ex Vivo* and *In Vivo* Analysis

The antigen dose delivered into the skin and the reproducibility of the antigen delivery can be determined in *ex vivo* or *in vivo* studies (63), either indirectly by measuring the remaining antigen in the dissolved MNs or directly by measuring the antigen in the skin. Direct quantification can be performed by using either radioactivity (91) or infrared imaging.

## Immunogenicity of Antigens Administered by dMNs: Preclinical Studies

The first successful vaccination with dissolving microneedles was reported in 2010 (55). Table III gives a summary of the reported immunization studies. Depending on the antigen, a humoral and/or cellular response is important for a therapeutic effect.

**Table III:** Immunization Studies with dMNs

Antigen/ Adjuvant (dose)	Animal model	Immunization site and application method	Immunization scheme	Immune response analyzed	dMNs result *vs* other groups	Ref.
OVA 1 μg	C57Bl/6 and Wistar ST rats	Back skin	4 times every 2 weeks	Ab response	IgG levels equal or superior to s.c. or i.d. group	(21)
		Handheld applicator				
OVA 10 μg, 100 μg	BALB/c mice	Dorsal skin	2 times every 2 weeks	Ab response	IgG levels comparable to i.d. group	(36)
		Manual application				
OVA 15 μg, 50 ng poly(I:C) in PLGA NPs	C57Bl/6	Dorsal ear skin	2 times every 35 days	Ab and T-cell response	IgG levels comparable to the i.m. and i.d. groups at day 63	(34)
		No applicator mentioned			CD8^+^ T-cells similar to i.d. groups and higher than i.m. after booster dose.	
					Central memory CD8^+^ T-cells higher than i.d. and i.m. groups	
OVA 9 μg poly(I:C) 150 ng	C57Bl/6	Dorsal ear skin	Single vaccination for dMNs	Ab and T-cell response	Both CD8^+^ and IgG response higher than with i.d. injection	(35)
		No applicator mentioned	Boost on day 28 for i.d. injection		Central memory CD8^+^ T-cells higher than i.d. group	
OVA 1 mg	Sprague Dawley (SD) rats	Back skin	Single vaccination	Ab response	IgG levels higher than i.m. group	(37)
		Homemade applicator				
OVA 2 μg and	BALB/c mice	Abdomen skin	2 vaccinations after 3 weeks	Ab response	IgG levels higher than i.m. group	(25)
CpG OND 10 μg co-encapsulated in cationic liposome		Homemade applicator				
PLGA NP-encapsulated OVA 10 μg	C57Bl/6	Dorsal ears skin	Single vaccination	T-cell response and challange	In dMN group: CD8+ T-cell response with central and effector memory profiles.	(28)
		Manual application			Growth of melanoma tumor through the Th1 IFN-γ mediated response suppressed	
					Protection against respiratory challenge with OVA-expressing virus	
OVA7.6 μg / Quil-A 0.2 μg (2 patches per mouse)	C57Bl/6	Ventral ear skin	Single vaccination	Ab response	IgG levels (lower dose than i.m.) higher after 102 days than i.m. group	(55)
OVA 0.4 μg / Quil-A 0.01 μg (1 patch per mouse)		Spring applicator			IgG levels (lower dose than i.m.) comparable after 102 days than i.m. group	
Split virus influenza vaccine 0.06 μg (1 patch per mouse)	C57Bl/6	Ventral ear skin	Single vaccination	Ab response	IgG levels (lower dose than i.m.) lower than i.m. group	(55)
Split virus influenza vaccine 0.12 μg (2 patch per mouse)		Spring applicator				
Inactivated Influenza Virus 6 μg	BALB/c mice	Dorsal skin	Single vaccination	Ab and T-cell response	IgG levels slightly lower (after 14 days) and then similar (after 28 days) than i.m. group	(26)
		Manual application			HAI similar to i.m. group	
					Cellular response similar to the i.m. route	
Inactivated split TIV 0.375 μg HA	BALB/c mice	Ear	Single vaccination	Ab response	Anti-HA IgG response higher than i.m. group	(38)
Inactivated split TIV 3 μg HA		Manual application			Anti-HA IgG comparable but more durable than i.m group	
					HI titers comparable to i.m. group	
Influenza vaccine H1N1 0.1 and 1 μg HA	BALB/c mice	Not reported	2 times after 4 weeks	Ab response	HI and IgG titers higher than i.m. group	(32)
					Microneutralization titers lower than i.m. group	
TIV 0.1 μg HA					HI titers after the boost lower than i.m. group	
Cell culture-derived influenza subunit trivalent vaccine 3 x 2.5 μg HA and 3 x 10.8 μg HA	Hartley guinea pigs	Dorsal skin	2 times after 3 weeks	Ab response	IgG and HI titers comparable to i.m. group	(39)
		Spring-based applicator				
H1N1 3 μg of HA	BALB/c mice	Dorsal skin	Single vaccination	Ab response	HAI, IgG and VNT higher than i.m. group	(40)
H3N2 3 μg of HA		Manual application			HAI titers higher than i.m. group	
					VNT and IgG titers similar to i.m. group	
B 3 μg of HA					HAI, IgG and VNT higher than i.m. group	
Ad type 5 - OVA vector (4.3 x 10^8^ VP)	C57Bl/6 and B6	Dorsal surface of the foot, ear or back skin	Single vaccination	T-cell response	SIINFEKL- specific CD8+ T-cells indistinguishable with i.d., s.c. and i.m. groups	(33)
Ad type 5 – HIV/gag vector (4.3 x 10^8^ VP)		Manual application			CD8+ T-cell frequencies comparable with i.d. group	
Ad (7.7 x 10^9^ VP)	Hairless rats	Back skin	3 times after 2 weeks	Ab response	IgG titers equal to s.c. group	(21)
		Handheld Applicator				
EV71 VLP 1 μg	BALB/c mice	Dorsal skin	3 times after 2 weeks	Ab, T-cell response and challenge	IgG and VNT comparable to i.m.(10 μg) and higher than s.c. (10 μg) after the third vaccination	(24)
		Applicator			100% survival after challenge	
					Stronger T-cell response than i.m. and s.c. (both 10 μg)	
IPV type 1 (47 D-antigen units)	Rhesus Macaques	Upper back skin	2 times after 8 weeks	Ab response	No difference in IgG responses with i.m. group	(30)
IPV type 2 (9 D-antigen units)		Manual application			No difference in IgG responses with i.m. group	
IPV type 3 (38 D-antigen units)					IgG lower than in the i.m. group.	
					This difference is due to a mistake in the IPV type 3 quantification: the real dose in the patch was 3x lower than 38 D-antigen units	
Divalent toxoid vaccine (TT 20 μg and DT 10 μg)	Wistar ST rats	Back skin	5 times after 2 weeks	Ab response	Both anti-TT and anti-DT IgG titers after dMNs stored vaccination comparable with those induced by freshly prepared dMNs.	(22)
		Handheld applicator				
Measles Vaccine (3100 TCID50)	Rhesus Macaques	Upper back skin	Single vaccination	Ab response	VNT titers equivalent to that of s.c. group	(31)
		Manual application				
Vector encoding the middle envelope proteins of HBV 10 μg	BALB/c mice	Abdominal skin	2 times after 3 weeks	Ab response	IgG comparable to i.m. group	(27)
CpG ODN 10 μg		Manual application				
Encapsulation (with or without Adj) in cationic liposomes						
DNA plasmid expressing rabies G protein 50 μg	Beagle dogs	Inner ear pinna	2 times after 4 weeks	Ab response	VNT titers comparable (42 days after the prime) and higher (56 days after the prime) than i.m. group	(41)
DNA plasmid expressing rabies G protein 5 μg		Application by thumb			VNT titers lower than i.m. group	
HIV-1 CN54gp140 10 μg MPLA (20 μg)	BalB/c	Ear	4 times after 2 weeks	Ab response	IgG titers lower than s.c. group	(29)
		Application by thumb				

*Ad* adenovirus, *B* Brisbane, *CpG ODN* CpG oligodeoxynucleotides, *DT* diphtheria toxoid, *EV71* Enterovirus 71, *HA* hemagglutinin, *HBV* hepatitis B virus, *HIV* human immunodeficiency virus, *HN* hemagglutinin and neuraminidase, *IPV* inactivated polio vaccine, *MPLA* monophosphoryl lipid A, *NPs* nanoparticles, *OVA* ovalbumin, *PLGA* poly-D,L-lactide-co-glycolide, *poly(I:C)* polyinosinic-polycytidylic acid, *TIV* trivalent influenza vaccine, *TT* tetanus toxoid, *VLP* virus like particles, *VNT* virus neutralization test, *VP* virus particles

### Animal Models and Application Method

Mice are the most frequently used animal model, particularly BalB/c (23,25–27,29,32,36,38,40) or C57BL/6 (21,28,33–35,55) strains. Transgenic T-cell receptor mouse models (e.g. OT-I mouse for examining CD8^+^ T-cell response) can be also used as immunological model (92–94). However, animal models with skin anatomy that mimics more closely human skin may be more relevant, for example guinea pigs for influenza (39), beagle dogs for rabies vaccination (41) and rhesus macaques for measles and polio vaccination (30,31).

The dMN patch can be applied either manually, particularly if MN length is over 500 μm, (26–31,33,36,38,40,41) or by using an applicator (21,22,25,37,39,55). The advantages of the manual application are simple administration and reduced costs (30). However, efficient skin piercing after manual application might be limited to longer MNs (>550 μm) while shorter MNs (300 μm) might require an applicator (95,96). Besides the penetration efficiency, an applicator improves the reproducibility of the piercing, that is expected to lead to a more reproducible delivery of the vaccine (97).

### Humoral Immune Response

#### OVA

The model antigen ovalbumin (OVA) is most commonly used in dMN immunization studies due to its relatively low cost and excellent stability (98) and the strong immunogenicity in mice. However, these beneficial characteristics mean that the results obtained with OVA may obscure formulation problems with more relevant vaccine antigens. Several studies with OVA-containing dMNs have shown that IgG responses are either equal or superior to the ones obtained by s.c., i.m. or traditional i.d. injection of the same dose (21,25,28,34,36,37,55). Furthermore, non adjuvanted OVA dMNs (10 μg) showed a higher response than topical application of cholera toxin-adjuvanted OVA (100 μg) on intact skin (36). This indicates the importance of a direct delivery of the entire antigen dose into the skin to induce an immune response.

In another study, OVA loaded chitosan dMNs elicited higher IgG response than i.m. injection of OVA solution after single immunization in rats. This can be explained by a gradual degradation of chitosan microneedles creating a depot effect in the skin (37). The OVA containing chitosan microneedles were mounted on a PLA support. After application, the chitosan microneedle tips were released from the support, forming a depot in the skin. Even two weeks after the dMN application, chitosan and OVA were still present in rat skin. Similarly, single immunization with cross-linked silk/poly(acrylic acid) (PAA) dMNs evoked higher IgG response than the i.d. injection of OVA (35). However, in this case sustained release from the cross-linked silk in the PAA dMNs (100% within 12 days) did not improve the response compared to fast release from PAA dMNs (100% within 6 days). (35). Similarly, single immunization with Quil-A adjuvanted OVA dMNs resulted in stronger long-lasting IgG response than Quil-A adjuvanted OVA after i.m immunization (55). Twenty-eight days after a single immunization, dMNs (dose 7.6 μg) had similar IgG response to i.m injection (15 μg) despite the lower dose. At day 102, the IgG response of dMNs (7.6 μg) was higher than that of i.m (15 μg), and even more interestingly low-dose dMNs (0.4 μg) had similar response to i.m. immunization (15 μg) (55). However, it must be noted that dMN patches were applied at two sites (both ears) while i.m injection was performed only at one site. Draining to two lymph nodes may have an effect on the magnitude of the response. Also, the ear is a very sensitive location for dermal vaccination probably for the short distance to one major draining lymph node (99).

The use of dMNs have been shown to affect the Th1/Th2 balance. Single immunization with cross-linked silk/PAA dMNs evoked strong IgG1 and IgG2c response while i.d. injection elicit only IgG1 response, and thus dMN immunization shifted Th1/Th2 balance toward Th1 (35). These results were supported by another study where hyaluronan-based OVA dMNs were compared to s.c. and i.d. injections in mice (21). In contrast, in rats no IgG2c response was detected neither after dMN, s.c., or i.d. immunization in the same study (21). Additionally, in another study the shift in Th1/Th2 balance was not observed after dMN immunization in mice (25). As conclusion, dMN vaccination may affect the Th1/Th2 balance but further studies are needed since the number of publications on this subject is limited.

#### Influenza

Immunization with influenza vaccine loaded dMNs resulted often in higher ((38) (40)) or comparable (26) IgG response than i.m. administration. However, Kommareddy *et al*. showed that dMNs evoked lower IgG response than i.m. immunization after the boost, although the response induced by dMNs was higher after the prime (32). However, in other studies contradicting results were found. Haemagglutination inhibition titers and antibodies and neutralizing antibody titers after the dMN immunization were similar (26) or superior (40) to i.m. immunization. Stabilization of the antigen by addition of sucrose (40) may have allowed to obtain a higher antibody titers than the previous work (26). Furthermore, the difference with the above mentioned study (32) could be explained by the use of a different assay (ELISA assay) than the one routinely used to investigate the influenza vaccine quality (single radial immunodiffusion (SRID) assay). Interestingly, the dry matrix of dMNs can stabilize the antigen up to one year in comparison to liquid formulation (38). In summary, most studies show that influenza vaccination by dMNs can evoke comparable or even superior responses than i.m. immunization.

#### Other Antigens

Different types of antigen, such as vector, live attenuated and inactivated vaccines, have been loaded in dMNs and evaluated *in vivo*. An example is the vaccination of rats with the model antigen adenovirus (Ad) loaded dMNs: Ad-specific IgG titers observed were comparable to the s.c. group, while topical application showed no IgG response (21). In a study examining the dose-sparing effect, mice were immunized with dMNs loaded with 1/10th the dose of Enterovirus71 (EV71) – virus-like particles compared to immunization with a full dose i.m. and s.c. injected vaccine. Antibody and neutralizing titers both revealed comparable responses to i.m. and higher responses than s.c. after the three immunizations. Furthermore, the dMN group, together with s.c. and i.m. groups, survived the lethal virus challenge showing the protective effect of the dMNs (24). Rhesus macaques were used as animal model to examine the immune response after vaccination with inactivated polio vaccine (IPV) (30) and live-attenuated measles vaccine (31). In both cases, neutralizing antibody titers after dMN immunization were comparable to that after s.c. (measles) and i.m. (IPV) immunization.

In the case of dMNs loaded with DNA containing the rabies G-protein gene, comparable neutralizing antibody titers with i.m. were detected after a booster. No evidence of the dose sparing in dMNs was found since the antibody titers of 10-fold lower dose were clearly weaker than those of full dose in dMNs (41). The co-encapsulation of plasmid vector against hepatitis B virus (HBV) and CpG in cationic liposomes in dMNs resulted in slightly higher IgG titers than free antigen and adjuvant in dMNs (27). It should be considered that the characteristics of liposomes changed after loading in dMNs (increase in size and decrease in Z-potential). However, the immune responses were generally similar between dMN and i.m immunization, and adjuvant and liposomes did not affect the IgG1/IgG2a balance (27).

### Cellular Immune Response

De Muth *et al*. have reported two studies in which dMN immunization elicited high CD8+ T-cell responses. Mice were immunized with dMNs made of fast-dissolving PAA containing OVA mixed with PLGA microparticles (size 1.6 μm) encapsulating poly(I:C) (34) or cross-linked silk structure of OVA and poly(I:C) (35). The latter results in a binary release profile: a burst of OVA after dMN dissolution followed by a sustained OVA release from the cross-linked silk structure. Both studies indicated that the CD8+ T-cells producing IFN-γ and TNF-α, upon peptide stimulation, are increased by dermal sustained release (>16 days) from dMNs in comparison with i.m. injection of sustained release of poly(I:C) from PLGA microparticles (34), or with i.d. injection of soluble OVA and poly(I:C) (35). Furthermore, when comparing the different dMNs, the sustained release of cross-linked silk/PAA microneedles additionally increased the CD8+ response in comparison with fast release of PAA microneedles (35). In addition, a prime immunization with dMNs can produce a similar fraction of functional CD8+ T-cells as a prime and boost with i.d. injection (35). Despite a larger effector CD8+ T-cell response, dMN delivery also resulted in a more rapid transition to central memory CD8+ T-cells than i.m. and i.d. injections, suggesting the additional expansion of CD8+ T-cells after dMN delivery did not solely result in more terminally differentiated effector cells (34) (35). However, sustained release from dMNs did not further improve the memory response (35). A long-term memory immune response was reported also after vaccination by Na-CMC dMNs loading recombinant adenovirus vector encoding HIV-1 gag. Vaccination by dMNs generated CD8+ memory T-cells comparable with the intradermal injection (100). Supporting results have been found also with other MN technologies inducing a better long-term memory response than s.c. (101) or i.d. (102) injections.

The PLGA NPs dMN concept may have potential for therapeutic cancer vaccination: dMN immunization suppressed the growth of melanoma tumor, evoked in mice by injecting OVA-tumor cells, through antigen-specific CD8+ T cells (28). Furthermore, OVA-PLGA NPs dMN immunization protected against respiratory viral challenge with a recombinant Sendai virus expressing OVA (28). The vaccine depot and particulate vaccines may induce a better T-cell immune protection because the response correlates with antigen persistence (103), the sustained antigen release (104) or particulate nature of vaccine. To elucidate the immunological mechanism, it was shown that Langerhans cells are required for cytotoxic CD8+ responses (28) (105). Langerhans cells apparently efficiently process the OVA loaded in the microparticles which leads to cross presentation by MHC class I molecules. To support this explanation, the role of Langerhans cells was less significant for soluble OVA compared to particulate OVA (105).

In another study with dMNs loaded with EV71 virus-like particles, vaccination by dMNs loading 10 times lower antigen dose than i.m. and s.c. injections could promote stronger EV71-specific T-cell response than the conventional injections (24).

Viral vectors are able to induce strong T-cell responses after dMN immunization. dMNs with human adenovirus expressing ovalbumin were compared to i.d, i.m. and s.c. injections. The T-cell responses were similar in all groups (33). Similarly, CD8+ T-cell responses were comparable after mice were immunized with rAdHu5 vector encoding a HIV-1 Gag gene by dMNs or i.d. injection (33).

### dMN Immunization: Factors Influencing the Immunogenicity

#### Adjuvants

Several adjuvants have been used in dMNs and they are similar to those used for other administration routes except aluminum based adjuvants and emulsions. Aluminium based adjuvants may cause local adverse effects like granuloma formation and therefore is not suitable for delivery to the skin (106). Emulsions cannot be formulated in dry formulations like dMN because water is a structural part of the formulation. Molecular immune modulators, such as CpG (25,27), poly(I:C) (34), Quil-A (55), monophosphoryl lipid A (MPLA) (29) and imiquimod (107) have been used in dMNs. In general, a significant increase in the immune response is observed when an adjuvant is included in dMNs (25,27), although sometimes the control group without the adjuvant is lacking. Unfortunately, the rationale of selection a certain adjuvant and its dose has not often been addressed.

Delivery systems can be formulated into dMNs (see previous section). Similarly to other administration routes, encapsulation of antigen (and adjuvant) in nanoparticles or liposomes can enhance the immune response after delivery with dMNs (25,27,28) as described above (Humoral Immune Response and Cellular Immune Response Sections). Adjuvants are often needed with modern subunit vaccines but their use might be avoided with attenuated viruses and viral vectors. Absence of adjuvant would also facilitate batch release since adjuvant quantification is not needed and antigen quantity is often limited to a simple plaque titration or colony count as opposed to an immunogenicity test in experimental animals.

#### MNs Spacing and MN Geometry

Modelling studies have indicated that the MNs spacing may affect the immune response by contributing to the optimal antigen concentration released into the skin to activate APC located between the MNs (108). However, this is not experimentally confirmed and factors not accounted for in the model may contribute significantly to the immunogenicity.

The MN length may influence the population of APCs activated so that shorter MNs could activate LCs in the epidermis and longer MNs could activate dDCs in the dermis (108). *In vivo* studies with 1 μg OVA showed that IgG response after vaccination by dMNs of 300 and 800 μm in length is higher than using dMNs of 200 μm in length (21). On the other hand, the variation of injection depth with hollow MNs did not affect the immune response (109). However, while a controlled antigen dose was released at different skin depth by hollow microneedles (109), not clear is the antigen dose released into the skin from the dMN of different lengths (21). This could explain the difference in the immune response.

Apart from MN length, needle density may be an important variable with respect to immunogenicity. The needles cause minor damage and cell death, initiating a pathway acting as “natural immune enhancer” mediated by the release of damage-associated molecular patterns (DAMPs) (110). In fact, the same antigen dose released by coated MNs elicited higher response than a single i.d. injection (110).

## Clinical Development of Dissolving MicroneedleS

dMNs are a relatively new vaccine delivery system with no licensed vaccines and few results from clinical studies. Two phase 1 (safety) studies with microneedles without antigen have been performed so far. In the first, hyaluronan microneedles (length 300, 500 and 800 μm, 200 dMNs on a 0.8 cm^2^ patch) have been applied on 17 subjects (53). Despite a successful dMN penetration into the skin by using an applicator, the microneedles required 6 h of application for nearly complete dissolution in all subjects, which may be too long for routine immunization. In the second study, PVA microneedles (length 650 μm, 100 dMNs on a 1 cm^2^ patch) have been applied on 15 subjects (111). In this case, an average of 100% piercing efficiency of MNs into the skin without any applicator use was reached. However, variance in the microneedle volume dissolved, especially among subjects using self-administration, underlined the importance of using an applicator to have a controlled force and an impact during application. Few subjects (53) or all of them (111) showed a slight erythema after dMN application that disappeared within 7 days. However, longer dMNs of 500 and 800 μm caused purpura, indicating capillary damage, in 50% of the volunteers but shorter 300 μm dMNs did not induce any purpura (53). No swelling at the application site (111) or systemic adverse events were observed (53). Additionally, it was also concluded that dMN application caused hardly (53) or no pain (111).

In another clinical phase 1 study, trivalent influenza hemagglutinins vaccination with sodium hyaluronate dMNs (800 μm, 200 dMNs on a 0.8 cm^2^ patch, spring-type applicator used) was investigated in healthy subjects (112). dMNs loaded with 3 x 15 μg of influenza antigens on a single patch, were compared with the same dose administered by s.c. injection. During the prime immunization a proper dMN dissolution was observed in only seven subjects out of 20 and only these subjects were included in the final analysis. Furthermore, the applicator settings were changed to obtained a more efficient application in the second vaccination. After the prime, the anti-HI antibody titers against influenza A HA-antigens (H1N1 and H3N2 strains) were equivalent in the dMN and s.c. groups, except that for the B strain that showed higher titers in the dMN group also observed in preclinical studies (32). More IFN-γ-producing peripheral blood mononuclear cells were detected after s.c. than dMN immunization (112). However, the low number of subjects in dMN group limits the conclusions. Regarding the safety, erythema detected in the dMN group was higher than the s.c. one and more pronounced than in the previous clinical studies (53,111). Purpura was observed in 50% of the subjects both in the dMN and the s.c. group. However, no adverse systemic events were observed (112).

These studies prove that the applicator and its settings have a crucial role for MN penetration and subsequent dissolution into the skin. Alternatively, encapsulation of the antigen only in the microneedle tip can enable a complete antigen delivery even with incomplete microneedle dissolution (e.g., localizing the antigen in the upper 70% of the MNs, a 70% dissolution would correspond to 100% antigen delivery).

Besides above mentioned studies, at least one other study has been performed to investigate safety and immunogenicity of influenza vaccination with dMNs but the results are not yet published (113).

## Conclusions and ProspectS

dMN vaccination can offer important advantages such as dose sparing, pain-free immunization and avoidance of needle-stick injuries. Furthermore, it can extend the vaccination coverage in developing countries by potentially offering improved vaccine stability, reduction of vaccine wastage and of burden on trained personnel. However, several improvements are still needed in some areas of dMN development before the regulatory acceptance and industrial scale-up are feasible. Fabrication methods require further optimization to enable the minimal wastage of antigen that is often claimed but rarely reported in the literature, and not yet proven on at least pilot scale production level. Analytical challenges include potency testing and stability testing during fabrication and storage, and quantification and reproducibility of antigen/adjuvant dose delivered in the skin. Furthermore, the role of the applicator device should not be underestimated because it can standardize dMN application and vaccine delivery into the skin, although with respect to logistics manual application is preferred. dMN immunization has generated comparable or higher and more durable antibody and cellular responses than conventional immunizations in preclinical studies. Additionally, sustained release of antigen from nanoparticles or cross-linked structures in dMNs showed to induce a better cellular immune response than fast release from dMNs or liquid solution, although the sustained release from dMNs did not improve further the humoral response than fast release from dMNs. However, further studies should be performed to support this conclusion. In the future, more systematic studies, such as identification of optimal adjuvants and analysis of effect of dMN geometry, may be necessary to optimize dMN immunization. Until now, three clinical phase 1 studies have been reported and showed that skin irritation and patch application are hurdles that need to be solved in future applications. The ideal dMN patch (Table IV) does not exist yet but encouraging progress has been made. More work is needed to further develop dMNs into safe, efficacious, affordable and widely used products.

**Table IV:** Target Product Profile of the Ideal dMN Patch

	Total systems costs lower than injected vaccine
	Competitive production costs
	Simple to produce
	Stable outside the cold chain
	Higher immunogenicity / dose sparing / single shot
	Minimum waste
No applicator needed	
Fail-proof application/check on full dose delivery	
Short application time	
Less adverse effects	
Affordable	

## Abbreviations

Ad: Adenovirus
AF4: Asymmetrical flow field–flow fractionation
APCs: Antigen presenting cells
B: Brisbane
CD: Circular dichroism
CpG ODN: CpG oligodeoxynucleotides
DAMPs: Damage-associated molecular pattern
DAB: Droplet-born air blowing
dDCs: Dermal dendritic cells
DEPA: Dissolving microneedles on an electrospun pillar array
DLS: Dynamic light scattering
dMNs: Dissolving microneedles
DT: Diphtheria toxoid
ELISA: Enzyme-linked immunosorbent assay
EV71: Enterovirus 71
FDA: Food and drug administration
HA: Hemagglutinin
HBV: Hepatitis B virus
HIV: Human immunodeficiency virus
HN: Hemagglutinin and neuraminidase
HP-SEC: Size exclusion chromatography
i.d.: Intradermal
i.m.: Intramuscular
IPV: Inactivated polio vaccine
LCs: Langerhans cells
MFI: Micro-flow imaging
MNs: Microneedles
MPLA: Monophosphoryl lipid A
Na-CMC: Sodium carboxymethylcellulose
NPs: Nanoparticles
NTA: Nanoparticle tracking analysis
OVA: Ovalbumin
PAA: Poly(acrylic acid)
PDMS: Polydimethylsiloxane
PGA: Polyglycolic acid
PLA: Polylactic acid
PLGA: Poly-D,L-lactide-co-glycolide
PMVE/MA: Methylvinylether-co-maleic anhydride (Gantrez® AN-139)
Poly(I:C): Polyinosinic-polycytidylic acid
PVA: Poly(vinylalcohol)
PVP: Poly(vinylpyrrolidone)
s.c.: Subcutaneous
SDS-PAGE: Sodium dodecyl sulfate-polyacrylamide gel electrophoresis
SRID: Single radial immunodiffusion assay
TEM: Transmission electron microscopy
TIV: Trivalent influenza vaccine
TT: Tetanus toxoid
UV-Vis: Ultraviolet–visible spectroscopy
VLP: Virus like particles
VNT: Virus neutralization test
VP: Virus particles
